# A study protocol of a randomised controlled trial to investigate if a community based strength training programme improves work task performance in young adults with Down syndrome

**DOI:** 10.1186/1471-2431-10-17

**Published:** 2010-03-25

**Authors:** Nora Shields, Nicholas F Taylor, Bo Fernhall

**Affiliations:** 1 School of Physiotherapy, La Trobe University, Melbourne, Victoria 3086, Australia; 2 Musculoskeletal Research Centre, La Trobe University, Melbourne, Victoria 3086, Australia; 3 Department of Kinesiology and Community Health, University of Illinois at Urbana-Champaign, 227 Freer Hall MC-052, 906 South Goodwin Ave, Urbana, IL 61801, USA

## Abstract

**Background:**

Muscle strength is important for young people with Down syndrome as they make the transition to adulthood, because their workplace activities typically emphasise physical rather than cognitive skills. Muscle strength is reduced up to 50% in people with Down syndrome compared to their peers without disability. Progressive resistance training improves muscle strength and endurance in people with Down syndrome. However, there is no evidence on whether it has an effect on work task performance or physical activity levels. The aim of this study is to investigate if a student-led community-based progressive resistance training programme can improve these outcomes in adolescents and young adults with Down syndrome.

**Methods:**

A randomised controlled trial will compare progressive resistance training with a control group undertaking a social programme. Seventy adolescents and young adults with Down syndrome aged 14-22 years and mild to moderate intellectual disability will be randomly allocated to the intervention or control group using a concealed method. The intervention group will complete a 10-week, twice a week, student-led progressive resistance training programme at a local community gymnasium. The student mentors will be undergraduate physiotherapy students. The control group will complete an arts/social programme with a student mentor once a week for 90 minutes also for 10 weeks to control for the social aspect of the intervention. Work task performance (box stacking, pail carry), muscle strength (1 repetition maximum for chest and leg press) and physical activity (frequency, duration, intensity over 7-days) will be assessed at baseline (Week 0), following the intervention (Week 11), and at 3 months post intervention (Week 24) by an assessor blind to group allocation. Data will be analysed using ANCOVA with baseline measures as covariates.

**Discussion:**

This paper outlines the study protocol for a randomised controlled trial on the effects of progressive resistance training on work task performance and physical activity for adolescents and young adults with Down syndrome. The intervention addresses the impairment of muscle weakness which may improve work task performance and help to increase physical activity levels.

**Clinical trial registration number:**

Australian New Zealand Clinical Trials Registry ACTRN12609000938202

## Background

Down syndrome is the most commonly identified genetic cause of intellectual disability [1] with over 1 million people with Down syndrome living worldwide. Physical impairments commonly associated with Down syndrome include a higher prevalence of heart defects, muscle weakness and hypotonia, and low cardiovascular fitness [2-5].

Muscle strength is important for young adults with Down syndrome. Due to their intellectual disability, their workplace activities typically emphasise physical rather than cognitive skills such as packing boxes of confectionery, sorting and cutting clothing, and assembling automotive parts [6]. These physical work tasks can be problematic for young adults with Down syndrome as they typically have muscle weakness, hypotonia, and low cardiovascular fitness [2,4,5]. Their upper [7] and lower limb [3] muscle strength is up to 50% lower than their peers with typical development and their peers with intellectual disability without Down syndrome [8]. Muscle weakness contributes to a reduced ability to perform activities of daily living [9], and can negatively impact their vocational and social development in the workforce [10-12] as well as their quality of life [13].

A systematic review [14] suggested progressive resistance training (PRT) improves muscle strength in people with Down syndrome. PRT is a form of strength training where relatively high loads are lifted for a relatively low number of repetitions before muscular fatigue, and the load is progressed as the person gets stronger [15]. PRT is regarded as the best way to improve muscle strength when implemented with sufficient intensity and progression of load [15]. PRT is an appropriate intervention for adolescents and young adults with Down syndrome as they can master the repetitive skills required by this form of exercise and it can be conducted in an integrated and social community setting. Participation in team sports can be difficult for the majority of this group as they do not possess the coordination or agility to keep up with their peers without disability.

Only 3 published trials [6,16,17] have investigated the effects of a stand-alone PRT training programme in people with Down syndrome. Two of these trials [16,17] found improved upper and lower-limb muscle strength with training but neither reported the effects of the programmes on functional activities. The findings of these two trials were also limited because neither employed an assessor who was blind to group allocation nor did they include a control group so the effects of the training might have been due to series effects. The third trial is the only published RCT to investigate the effect of a stand-alone PRT programme and found it significantly improved upper limb muscle endurance for adults with Down syndrome (mean difference chest press repetitions at 50% 1 RM was 16.7, 95% CI 7.1 to 26.2, p = 0.002) [6]. The 10-week community based PRT programme was compared with usual care in 20 adults with Down syndrome (13 men, 7 women; mean age 26.8 ± 7.8 years) and also observed improvement in upper limb muscle strength (mean difference, chest press 1 RM 8.6 kg, 95% confidence interval -1.3 to 18.5 kg, p = 0.08) and upper limb functional activity at 10 weeks (mean difference grocery shelving task -20.3 s, 95% CI -45.7 to 5.2 s, p = 0.11) that approached but did not reach statistical significance. A limitation of this trial [6] was that the small sample size meant that it lacked power to detect clinically significant differences between the groups.

Research on barriers and facilitators for exercise has found people with Down syndrome require close supervision from a support person to participate in a high intensity exercise programme. Supervision is necessary to ensure they exercise at the correct intensity, to provide physical and motivational support and to help keep them focused [18]. Similar findings have been reported in studies on aerobic training for people with Down syndrome where constant encouragement was necessary to keep the participants going, as if left to themselves they would stop immediately especially if there were any distractions or diversions [19]. Social interaction has also been identified as the main reason people with Down syndrome participate in exercise. Similar themes have been identified by other researchers [20].

Improving community participation in exercise is a priority for young people with Down syndrome as they typically do not participate in the recommended levels of activity. A recent study suggests 75% of adolescents with Down syndrome do not meet the Australian guidelines on activity levels compared to 15-25% for their peers with typical development [21]. Muscle weakness is a specific impairment that contributes to a low level of fitness and a reduced ability to complete daily activity in people with Down syndrome [9]. Therefore, apart from the effect of muscle weakness on meaningful activities like work task performance, a related and important issue for people with Down syndrome is the extent to which their muscle weakness contributes to their low level of physical activity.

While an increase in muscle strength might be expected to improve the amount of physical activity they undertake, no studies have investigated this to date. Strength in people with Down syndrome has been associated with higher levels of physical activity. A study of people with Down syndrome who trained for Special Olympics events an average of 4.9 hrs per week had significantly greater isometric muscle strength of the lower back and limbs compared to people with Down syndrome who were sedentary. Decreased muscle strength is believed to impact on the ability of young adults with Down syndrome to perform everyday activities, including walking and running [22]. Therefore, improving muscle strength could potentially increase the amount of activity they undertake.

Implementing an exercise programme during adolescence is advantageous. Good exercise habits established early in life are important predictors of healthy activity patterns in adulthood [23,24]. Children with Down syndrome become less active during adolescence [25]. Their low levels of activity is concerning because of its association with obesity, type 2 diabetes, osteoporosis and cardiovascular disease [26]. Over 80% of people with Down syndrome are overweight [27] and people with Down syndrome are 10 times more likely to die from diabetes than the general population [28]. Therefore, it is important that young adults with Down syndrome are encouraged to engage in physical activity and exercise [29].

A student-led community-based PRT programme for young adults with Down syndrome is innovative and provides the supervision and social interaction people with Down syndrome need to exercise. Typically developing adolescents often exercise at a gym [30] and so this is a reasonable recreation option for adolescents with Down syndrome. Physiotherapy students make the ideal support people for adolescents with Down syndrome as they have an understanding of the principles of exercise training, and are close enough in age that the social relationship between the pair is meaningful. An additional beneficial outcome of this arrangement is that physiotherapy students have an opportunity to gain a unique experience of disability, something that is often missing from their professional training due to a lack of appropriate placements.

In summary, the published literature provides evidence that PRT can improve muscle strength and endurance in people with Down syndrome. There is now a need for a fully powered randomised controlled trial to investigate the effects of PRT on work task performance and level of physical activity for young adults with Down syndrome. We hypothesise that a community-based strength training programme will improve work task performance in adolescents and young adults with Down syndrome. The primary aim of this study, therefore, is to investigate if a community-based strength training programme can improve work task performance in adolescents and young adults with Down syndrome. The secondary aims are to investigate: (a) if the exercise programme leads to an improvement in muscle strength, and (b) if the exercise programme leads to an increase in the amount of moderate and vigorous level physical activity undertaken by adolescents and young adults with Down syndrome.

## Methods

### Research design

This randomised controlled trial will compare the effect of a 10-week student-led community-based PRT programme compared with a social programme. Specifically, this trial will examine if a student-led PRT programme improves work task performance and muscle strength in adolescents and young adults with Down syndrome and if it leads to improved activity levels after the programme has been completed. Seventy adolescents and young adults with Down syndrome will be recruited.

The trial has received ethics approval from the La Trobe University Human Ethics Committee and from the Victorian Department of Education and Early Childhood Development. Written informed consent will be sought from the next of kin (parent or guardian) of all adolescents with Down syndrome aged 14 to 17 years. The adolescents with Down syndrome will also be invited to provide their own written assent to participate in the study. For young adults with Down syndrome aged between 18 and 22 years, competence to give consent will be determined in conjunction with their next of kin. Where a young adult usually provides their own consent, they will provide their own informed consent for participation in this study. Where a young adult has been determined by their next of kin to not be cognitively able to provide their own consent, informed consent will be sought from the next of kin. In this case, the young adult will also be invited to provide their own written assent.

All participants will complete a baseline assessment after which they will be randomly allocated to either the intervention or control group. Following a 10-week period of training for the intervention group and a programme of social activities in the control group, all participants will be reassessed at Week 11. All participants will then continue with their usual activities for 3 months; at Week-24 both groups will complete a follow-up assessment to establish if any of the benefits of the programme have been maintained.

Each adolescent with Down syndrome will be paired with a student-mentor with whom they will exercise if allocated to the intervention group or complete a programme of social activities and hobbies including arts and crafts if allocated to the control group. All mentors will be physiotherapy students with an understanding of the principles of therapeutic exercise, and will receive instruction on how to complete either the progressive resistance training or the social programme. This feature will provide the supervision and social support needed to facilitate participation by the adolescents and young adults with Down syndrome in the exercise programme.

### Participants

Participants are adolescents and young adults with Down syndrome and will be recruited through local disability community groups and through specialist schools. Participants are eligible for inclusion in the study if they meet each of the following criteria:

• Young adults with Down syndrome aged 14-22 years, as this is the optimal age to implement an exercise programme within a community participation framework [20].

• Mild to moderate intellectual disability, based on carer report, as young adults with severe intellectual disabilities may have difficulty taking part.

• Ability to follow simple verbal instructions in English.

• Fit and well enough to participate in a high intensity PRT programme. All participants will complete the Physical Activity Readiness Questionnaire [31]. If indicated by the questionnaire, they will be asked to get medical clearance before taking part in the programme.

Participants are excluded from the study if they meet any of the following criteria:

• Participation in a PRT programme in the 3 months prior to their participation in the trial, to ensure that any effects can be attributed to the current intervention.

• A concurrent medical condition, such as chronic juvenile arthritis, autism, unrepaired congenital heart defect or Eisenmenger's complex in addition to a diagnosis of Down syndrome that might impact on their ability to participate in an exercise programme.

• A history of violent outbursts, absconding, aggressive behaviour or antisocial behaviour such as removing clothing in public.

### Randomisation

Participants will be randomly allocated to either the intervention or the control group using a concealed allocation, block randomisation method [32]. Participants will be considered in blocks of 4, 6 and 8. The order of the blocks will be generated from a random-number table and assignments sealed in sequentially numbered opaque envelopes. Only after the recruiter determines a participant is eligible for the study, the participant agrees to participate and they are enrolled, will assignment to group be made by opening the next envelope in the sequence. A member of the research team who is not involved in participant recruitment or training will be responsible for preparing and opening the envelopes.

### Intervention

Participants allocated to the intervention group will complete a 10-week, twice a week student-led PRT programme at their local community gym. A 10-week programme duration was chosen as physiologic training effects are evident after this time period in people with Down syndrome [14]. Each participant will attend their local gym twice each week with their student-mentor and both will complete the training programme. A community gym setting provides ready access to the equipment needed to perform this type of exercise training. All exercises will be conducted on pin-loaded weight machines as they are safer for novice participants than free weights as there is less chance of a weight being dropped on a body part causing injury. The student-mentor will be provided with a log book to record the details of each training session, including exercises completed, the weight lifted, the number of repetitions, and number of sets. In addition, the log book will have a section where the student will record details of any injuries or problems (adverse events) and any missed sessions. Each student-mentor will have contact with a member of the research team every 2 weeks to ensure training is consistent and proceeding as planned, to help address any issues with training that arise and to check the training logbooks are maintained and up-to-date. There will be some flexibility in the scheduling of sessions to make up for missed sessions due to illness or scheduling issues.

The participant and student-mentor will complete a PRT programme for the major muscle groups of the body. The programme will consist of 7 exercises, 3 for the upper body (for example, lat pull down, seated chest press, seated row), 3 for the anti-gravity support muscles of the lower body (for example seated leg press, knee extension, seated calf raise) and 1 trunk exercise. It will take approximately 45 minutes to 1 hour to complete. The training programme will conform to the principles of PRT as outlined by the American College of Sports Medicine [15]. Participants will complete 3 sets of 12 repetitions of each exercise, at a training intensity of 12 RM (that is, only 12 consecutive repetitions of each exercise can be completed before muscle fatigue is reached). This intensity of training is approximately equal to training at an intensity of 60-80% of one repetition maximum and provides a sufficient stimulus to increase the ability of muscle to generate force [15]. The weight lifted during an exercise will be increased when the participant can perform 3 sets of 12 repetitions of that exercise. Two-minute rest periods will be given between each exercise set.

### Student Mentors

The student mentors will be undergraduate physiotherapy students with an understanding of the principles of therapeutic exercise, and will be recruited through the School of Physiotherapy, La Trobe University. Information about the study will be provided during student forums and advertising flyers will be placed on notice boards. Students will be selected based on their geographical location; as the training will be conducted at community gyms, student mentors and young adults with Down syndrome who volunteer for the study will need to live in the same locality. We will also select a student mentor based on gender in the few cases where families indicate this is their preference.

To ensure consistency, student mentors will receive training on the content of the programme they will undertake. For example, if allocated to the PRT group, they will receive training on the PRT programme including programme progression, motivational and teaching strategies and how to use the gym equipment. The student mentor will also complete the exercise programme as our previous study found people with Down syndrome are far more likely to exercise when a support person joins in the activity with them [18]. The peer mentor will complete their set while the adolescent is resting and will use strategies such as counting to involve the adolescent in their programme.

### Control group

Participants in the control group will complete a social programme once a week for 10 weeks with a student mentor. This is to control for the social aspects of the intervention. The programme will comprise arts and recreational activities that would not be expected to have a fitness or training effect, such as painting, printing, music or social activities that do not incorporate a physical activity component. The exact content of the control group programme will be decided in consultation with the adolescent or young adult with Down syndrome and their family, so that the focus of the programme matches their goals and interests. Each session of the social programme will run for 90 minutes, approximately equal to the time spent in the 2 gymnasium sessions each week in the intervention group. The sessions will take place either in the participant's home or at a suitable local venue such as a library or community centre.

### Outcome measures

All outcome measures will be assessed at baseline (Week 0), immediately post intervention (Week 11) to establish the effects of the programme and 3-months post-intervention (Week 24) to determine if the benefits of the intervention are maintained. All assessments will be completed by an assessor who is blind to group allocation and who has no involvement in recruitment, the randomisation procedure, or the training programme.

#### Work task performance

The effect of the PRT programme on work task performance will be assessed using two tests (a) repetitive weighted box stacking and (b) weight carry test (pail carry). The repetitive weighted box stacking test requires the participants to repetitively lift 10 kg boxes, from the floor to a table 75 cm off the ground. The number of boxes stacked in one minute is measured. The weighted pail carry requires participants to carry two 20 litre buckets each weighing 10 kg around an oblong 10 m course marked with cones. The total distance covered in 30 seconds is measured in metres. Running is not permitted for safety reasons. These measures are recommended by the American College of Sports Medicine [33] and have demonstrated changes in people with intellectual disability [34,35].

#### Muscle strength

The effect of the PRT programme on muscle strength will be assessed by the amount of weight lifted in a single chest press and leg press (1 RM). These tests have been performed with high levels of retest reliability (r > 0.89) and have demonstrated no systematic change when an adult with neurologic impairment is not training [36].

#### Physical activity

The effect of the PRT programme on increasing physical activity levels will be measured with an RT3 activity monitor. This is a lightweight accelerometer worn on the participant's waistband. It monitors the frequency and duration spent in sedentary, moderate and vigorous level activity. It will be worn over 8 consecutive days (1 day familiarisation, 7 days data collection); this is sufficient time to obtain a reliable estimate (r = .76 to .86) of usual activity in adolescents [37]. The RT3 has demonstrated excellent intermonitor reliability (r = 0.99) [38] and its output is strongly correlated with oxygen consumption values (SVO_2_) [39,40]. These units have previously been used to measure physical activity in Australian adolescents with Down syndrome [25]. Accelerometers will be provided and fitted at each assessment and returned to the researchers by post. Parents or guardians will complete a short logbook to document the activities performed while wearing the accelerometer. This will assist in interpretation of the physical activity data.

### Data analysis

#### Sample size estimation

Sample size is based on a predetermined difference in performance of a work task outcome measure (weighted box stacking) between the intervention and control group of 25% (equivalent to an increase of 1.27 weighted boxes stacked per minute). This is based on the amount of difference that is believed to be clinically important. Sample size calculations assumed a standard deviation of 1.75 boxes per minute [35] and indicated 35 participants would be needed in each group for power of 0.8, assuming 10% of participants would be lost to follow up.

#### Statistical analysis

Data will be analysed using ANCOVA with the baseline score added as the covariate [41], with an alpha level of .05. Separate ANCOVAs will be completed to determine if the intervention group improved more than the control group at the end of training (at Week 11), and to determine if the intervention group had improved more than the control group after training had stopped for 3 months (at Week 24). The mean difference within group and the mean difference between groups and the associated 95% confidence intervals will also be calculated. To avoid bias and to maximize the randomization process, intention to treat analysis will be used. Where data are missing, the carry forward technique will be used, which assumes missing data remain constant [42].

## Discussion

This paper outlines the study protocol for a randomised controlled trial on the effects of progressive resistance training for adolescents and young adults with Down syndrome. It will establish if a novel, student-led community based PRT programme can improve work task performance and physical activity levels of young adults with Down syndrome. The trial is novel as it incorporates a student-mentor component designed to overcome some of the difficulties associated with participation in exercise for people with Down syndrome and encourages community participation as the training will take place at a local facility. The student mentor approach provides the programme with a potentially sustainable way of providing supervision for adolescents with Down syndrome when exercising.

If successful, PRT could be a feasible, meaningful, enjoyable and realistic recreation option for adolescents with Down syndrome as they make the transition to adulthood. This initial period of training may also give them the confidence to continue exercising after the trial finishes. The intervention addresses the impairment of muscle weakness which is likely to diminish their work task performance. It also has the potential to increase their physical activity levels in the short term and to decrease their risk of the secondary health consequences of inactivity in the long term.

This trial has the potential to make a significant difference to the health and employability of the more than 1 million people with Down syndrome worldwide. The trial outcomes will also impact on clinical practice by providing high level evidence to health professionals who work with people with Down syndrome about whether exercise programmes are effective for this group. If young adults with Down syndrome do become stronger and more physically activity, it may enable them in the short term to be more active in their school, work and home communities. The trial may also have long-term impact on the health of adults with Down syndrome, by providing them with an exercise option that will improve their work performance and lessen their risk of developing the secondary health consequences of inactivity.

## Abbreviations

CI: Confidence Interval; PRT: Progressive resistance training; RM: Repetition Maximum

## Competing interests

The authors declare that they have no competing interests.

## Authors' contributions

NS reviewed the literature, designed the study, and drafted the manuscript. NFT and BF made substantial contributions to the study design and contributed to the writing of the paper by revising it critically for important intellectual content. All authors read and approved the final manuscript.

## Pre-publication history

The pre-publication history for this paper can be accessed here:

http://www.biomedcentral.com/1471-2431/10/17/prepub
